# Supplementary material for: Salmonella typhimurium Vaccine Candidate Delivering Infectious Bronchitis Virus S1 Protein to Induce Protection
Source: Biomolecules. 2024 Jan 20;14(1):133. doi: 10.3390/biom14010133 (PMC10813627; doi:10.3390/biom14010133)
Supplement: Supplementary file 1 [file biomolecules-14-00133-s001.zip › biomolecules-2760943-original image.v1.pdf]

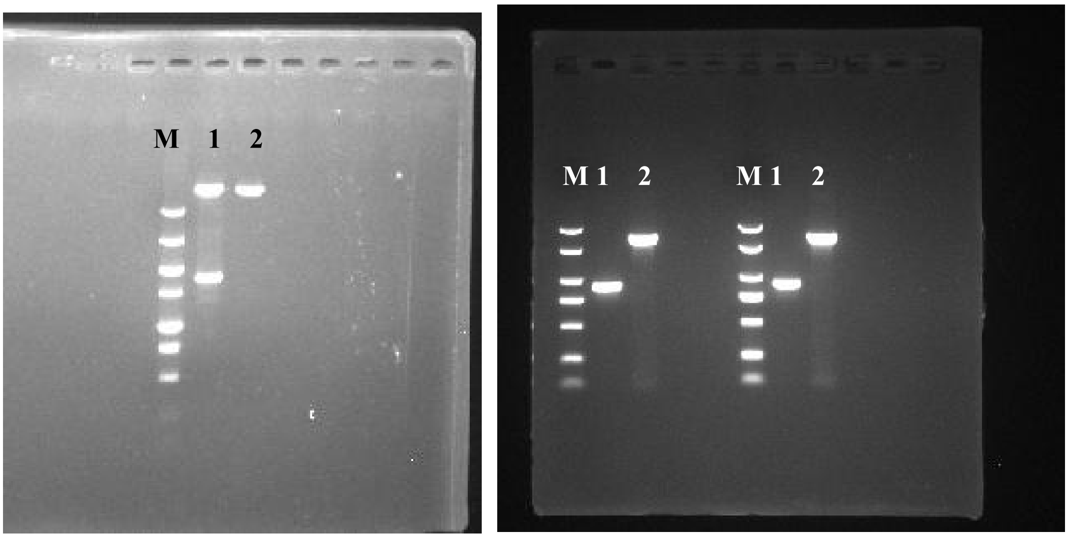

Fig. S1 **(C)** Identification of recombinant plasmid pYA4545-S1 by double enzyme digestion KpnI and XhoI.M: DL5000 DNA marker; Lane 1: pYA4545-S1 (1620 bp and 6953 bp); Lane 2: pYA4545. (**D)** The identification of delayed attenuation and lysis *Salmonella* χ11246. M: DL2000 DNA marker; Lane 1: The Δ*sifA* gene of χ11246 (850 bp); Lane 2: The *sifA* gene of UK-1 wild-type χ3761 (1683 bp).

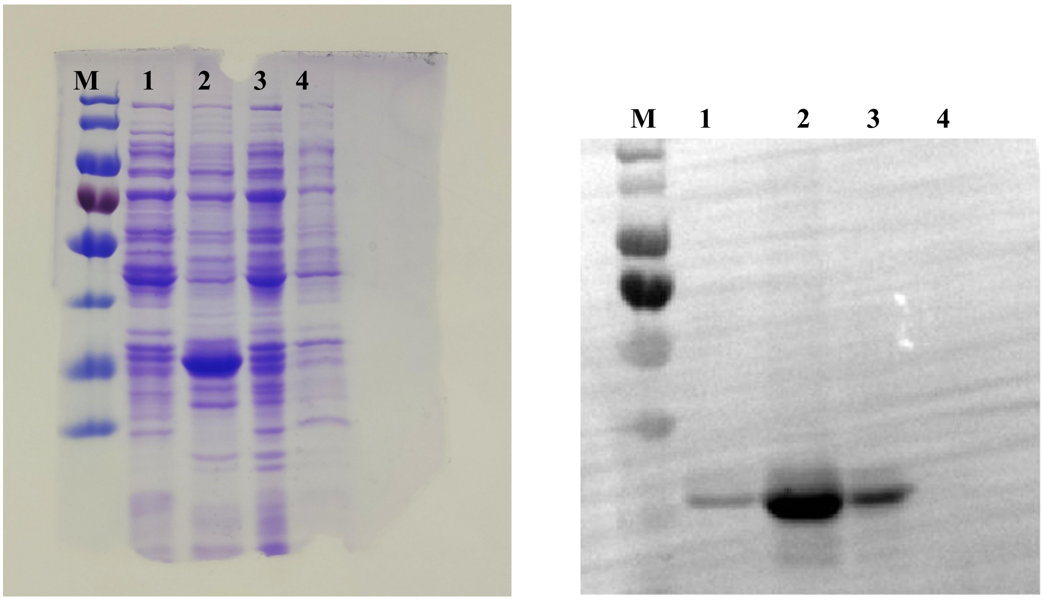

Figure S2. Expression of recombinant S1 protein. **(A)** SDS-PAGE analysis. **(B)** Western blot analysis. M: 180 kDa prestained protein marker; Lane 1: The supernatant of BL21(pET28a -S1); Lane 2: The precipitate of BL21(pET28a -S1); Lane 3: Positive control BL21(pET28a-GAPDH); Lane 4: Negative control BL21(pET28a).

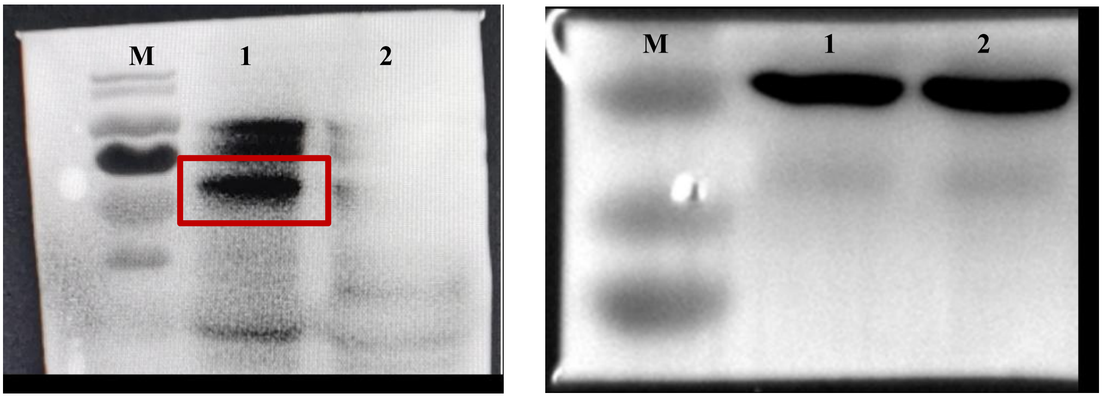

Fig. S3 **(C)** Western blot showed cells transfected with pYA4545-S1 had specific band while cells transfected with backbone plasmid pYA4545 had not. M:180 kDa prestained protein marker Lane 1：Transfected with pYA4545-S1; Lane 2：Transfected with pYA4545.
